# Evaluation of IL-2 and Dexamethasone intracavitary injection on the management of malignant effusion in children with solid tumors or lymphoma

**DOI:** 10.1186/s12885-021-09041-7

**Published:** 2021-12-06

**Authors:** Yu-Tong Zhang, Xiao-dan Zhong, Yan-li Gao, Jian Chang

**Affiliations:** 1 Department of Pediatric Oncology, The First Hospital of Jilin University, Changchun, Jilin, 130021 China; 2 Department of Pediatric Ultrasound, The First Hospital of Jilin University, Changchun, Jilin, China

**Keywords:** Pleural effusion, Ascites, Pericardial effusion, Interleukin-2, Pediatric cancer

## Abstract

**Background:**

Currently, no available coherent management protocol exists for pediatric cancers associated with pleural effusion, ascites, and pericardial effusion. This study aimed to retrospectively present our experience in treating pediatric cancer patients with pleural effusion, ascites, and pericardial effusion using interleukin-2 (IL-2) and dexamethasone (DEX) intracavitary injections.

**Methods:**

Between January 1st, 2008 and December 31st, 2020, medical reports of patients diagnosed with solid tumors or lymphoma were checked to identify patients diagnosed with > 2 cm pleural effusion, and/or more than grade 1 ascites, and/or more than small pericardial effusion. Patients diagnosed with effusions and treated with IL-2 and DEX were identified as being in the effusion group. Meanwhile, patients with the same primary tumors and effusions but did not receive interleukin 2 and DEX injection were reviewed and classified as the control group.

**Results:**

Forty patients with solid tumors and 66 patients with lymphoma were further diagnosed with pleural effusion, ascites, or pericardial effusion. A total of 85 patients received IL-2 and DEX injection while the remaining 21 did not. The Kaplan Meier analysis revealed a significant difference between the two groups, with *p* < 0.01 for event free survival (EFS) and *p* < 0.01 for overall survival (OS), both of which had *p* < 0.01. Hazard ratio was found to be 0.344 for OS and 0.352 for EFS.

**Conclusions:**

This retrospective study illustrates that thoracic, intraperitoneal, or pericardial intracavitary injection of DEX plus IL-2 can be an effective and safe treatment for pediatric cancers with pleural effusion, ascites, and pericardial effusion.

**Supplementary Information:**

The online version contains supplementary material available at 10.1186/s12885-021-09041-7.

## Introduction

Malignant pleural effusion, ascites, and pericardial effusion are common complications of most pediatric cancers, including 50% of patients with lymphomas and 50% with other tumors (like all kinds of sarcomas, neuroblastoma (NB), and hepatoblastoma (HB)) [1–3]. Malignant pleural effusion and pericardial effusion can cause breathlessness and are sometimes life-threatening. Moreover, hemorrhagic pleural effusion or ascites from a ruptured tumor may increase metastasis risk. As shown in a report from Children’s Oncology Group (COG) protocol AHOD0031, pleural effusion is an independent risk factor for the relapse of Hodgkin lymphoma [3]. However, no coherent management protocol is currently found for pleural effusion, ascites, and pericardial effusion. It seems that pleural effusion, ascites, and pericardial effusion can only be resolved with systemic chemotherapy, which can not only immediately relieve the discomfort of children but also increase metastasis risk. Several studies suggested that recombinant IL-2 may be effective for pleural effusion due to the ability to activate lymphokine-activated killer cells, which could induce a cytologic response to malignant pleurisy [4, 5]. Moreover, human natural killer cell activity is suppressed by asbestos fibers but could be restored by IL-2 in virto [6]. Since IL-2 amplifies and activates lymphokine-activated killer cells, This study retrospectively presents our 12-year’s experience in treating pleural effusion, ascites, and pericardial effusion with interleukin-2 (IL-2) and dexamethasone (DEX) intracavitary injections among pediatric cancer patients.

## Methods

This work was a single-center, retrospective cohort study. The ethics committee of our hospital approved our study protocols. Medical records from patients confirmed with solid tumors or lymphoma between January 1st, 2008 and December 31st, 2020, were reviewed to identify patients diagnosed with > 2 cm pleural effusion, and/or more than grade 1 ascites, and/or more than small pericardial effusion. For pleural effusion, small effusion (SE) is described as any effusion measuring 2–3 cm in size. Moderate effusion (ME) is any effusion > 3 cm in size that reached the mid-thoracic level on computer tomography (CT) image. Large effusion (LE) is any effusion that extends from the lung base to the apex and displaces heart and mediastinum toward the opposite side [3]. For ascites, grade 1 (G1) is mild ascites only detectable by ultrasound, grade 2 (G2) is moderate ascites evident by moderate symmetrical distension of abdomen, grade 3 (G3) is large or gross ascites with marked abdominal distension [7]. For pericardial effusion, total effusion (sum of the anterior and posterior) is categorized as small (S, 1 to 9 mm), moderate (M, 10 to 19 mm), or large (L, 20 mm or more) [8].

Patients diagnosed with effusions who received IL-2 and DEX injection were classified as effusion group. Meanwhile, patients with the same primary tumors with effusions who did not obtain any intracavitary injection were reviewed and classified as a control group. However, drainage was performed if a life-threatening breathlessness occurred due to effusions. To diagnose solid tumors or lymphoma, a fine needle biopsy or open biopsy was performed routinely.

The following patient data were extracted, including age, gender, tumor type, tumor stage, clinical manifestations of pleural effusion, ascites and pericardial effusion, therapeutic regimens of pleural effusion, ascites, and pericardial effusion, treatment response, and patient outcome. The Institutional Review Boards approved the collection of patients’ clinical records. All data were anonymous, and informed consent was waived due to retrospective observational nature of this study. For patients whose disease was measurable by CT or magnetic resonance imaging (MRI), their tumor responses were assessed according to revised-RECIST criteria. Complete response (CR), partial response (PR), progressive disease (PD), and stable disease (SD) were recorded accordingly [9].

### Treatment

While all patients in the effusion group were diagnosed with a malignant tumor and pleural effusion, ascites, or pericardial effusion, IL-2 and DEX injection therapy may be administered without a pathological diagnosis as long as malignant lesions associated with pleural, abdominal, or pericardial effusion were identified on imaging. We obtained written informed consent from patients’ parents or legal guardians before starting the therapy. First, patients performed thoracic, abdominal cavity, or pericardial cavity puncture with the indwelling of a drainage tube, and pathology was simultaneously obtained if permitted.

In patients with unilateral pleural effusion, no more than 600 mL fluid was drained on the first day and no more than 1000 mL each day; for patients with bilateral pleural effusions, the total drainage volume was the same. Ascites should not exceed 1000 mL each time, and pericardial effusion should not exceed 100 mL each time. In the presence of multi-cavity effusions, the drained effusion amount should be reduced as appropriate, and static electricity of hydration solution should be applied simultaneously.

After discharge, 0.9% sodium chloride injection (0.9%NaCl, maximum 100 mL) combined with IL-2 (5.0–10.0 × 10^6^ IU/m2, maximum dose 10.0 × 10^6^ IU) and DEX (5 mg) were injected via the drainage tube. The injection was administered every other day, and the total number of injections was not strictly limited, which was stopped when ultrasound confirmed that the pleural effusion or ascites was no more than 2 cm or disappeared in 24 h. The injection was stopped when total effusion of pericardial effusion was no more than S in 24 h. For patients with bilateral pleural effusions or multi-cavity effusions, the maximum total doses of IL-2 and DEX were maintained at 10.0 × 106 IU and 5 mg, respectively, which should be divided according to 0.9%NaCl volume. It should be noted that 0.9%NaCl volume should not exceed 50 mL during unilateral thoracic injection and should not exceed 20 mL during pericardial cavity injection. Besides, the injection time should be more than 1 h. It was advisable to use an injection pump to pump the fluid at a uniform rate. No strict requirement was found for the intraperitoneal injection rate or the fluid amount. After injection, the drainage tube was closed until the following day, and the child was instructed to change the position as much as possible to ensure the wider distribution of IL-2.

Chemotherapy might be initiated during IL-2 and DEX therapy. All patients in the control group were treated according to pathology diagnosis without IL-2 therapy.

#### Statistical analysis

The primary outcome was event-free survival (EFS) and overall survival (OS) rates. EFS was defined as the interval between diagnosis and disease progression, relapse, or death, and OS was defined as the interval between diagnosis and death from any cause or last contact. The Kaplan and Meier approach was used to estimate patient survival times. Kaplan Meier analysis was used to describe the time from IL-2 and DEX exposure to follow-up, and the log-rank test was used to compare findings between effusion and control groups. *P* < 0.05 was considered as significance difference. GraphPad Prism 8.0 was used for all statistical analyses and images. All proportions will be presented with 95% CI.

## Results

### Patients

Between January 1st, 2008 and December 31st, 2020, 372 patients were diagnosed with solid tumors, while 416 were diagnosed with lymphoma. Among them, 40 patients with solid tumors and 66 patients with lymphoma were further diagnosed with pleural effusion, ascites, or pericardial effusion. A total of 85 patients received IL-2 and DEX injection while the remaining 21 patients did not receive any drainage procedures. Indeed, 21 patients were diagnosed at early stage of this retrospective study. IL-2 and DEX injection were not routinely used to treat effusions at the time.

Among 85 patients in the effusion group, 21 had stage III diseases (including T cell lymphoblastic lymphoma, B cell lymphoblastic lymphoma, primitive neuroectodermal tumor (PNET), HB, and pediatric pneumoblastoma (PPB), respectively), while the remaining 64 had stage IV diseases (including T cell lymphoblastic lymphoma, B cell lymphoblastic lymphoma, diffuse large B-cell lymphoma (DLBCL), Burkitt’s lymphoma (BL), anaplastic large cell lymphoma (ALCL), rhabdomyosarcoma (RMS), NB, Ewing’s, HB, PNET, and PPB, respectively).

Accordingly, for patients without any intracavitary injection, 1 had stage II disease (HB in one), and one had stage IVs disease (NB in one). Nine cases had stage III diseases (including T cell lymphoblastic lymphoma, B cell lymphoblastic lymphoma, Ewing’s sarcoma, PNET and HB), while the remaining 10 patients had stage IV diseases (including T cell lymphoblastic lymphoma, B cell lymphoblastic lymphoma, DLBCL, ALCL, RMS and PNET). The detailed characteristics of 100 and six patients are presented in Table 1.

**Table 1:** The detailed characteristics of 106 patients

	Effusion group		Control group	
	Solid tumor	Lymphoma	Solid tumor	Lymphoma
Age (mean)	5.42	8.02	4.96	9.8
Gender				
Male	15	32	6	6
Female	14	24	5	4
Stage				
II			1	
III	4	15	5	4
IV	24	44	4	6
IVs			1	
Histology				
RMS	7		3	
NB	4		1	
PPB	3			
Ewing’s sarcoma	4		2	
HB	6		3	
PNET	5		2	
T cell lymphoblastic lymphoma		36		5
B cell lymphoblastic lymphoma		9		2
DLBCL		6		2
BL		3		
ALCL		2		1

*RMS* rhabdomyosarcoma, *NB* neuroblastoma, *PPB* pediatric pneumoblastoma, *HB* hepatoblastoma, *PNET* primitive neuroectodermal tumor, *DLBCL* diffuse large B-cell lymphoma, *BL* Burkitt’s lymphoma, *ALCL* anaplastic large cell lymphoma

Among patients who received IL-2 and DEX Injection, 58 patients only had pleural effusion (including bilateral pleural effusions in 29 patients), while 15 only had ascites. The remaining 12 cases had concurrent pleural effusion, ascites, or pericardial effusion, all of which had bilateral pleural effusions. For patients without any intracavitary injection, 11 patients only had pleural effusion (including bilateral pleural effusions in seven patients), while six only had ascites. The remaining four cases had concurrent pleural effusion, ascites, or pericardial effusion, all of which had bilateral pleural effusions. The detailed characteristics of patients with effusions are presented in Supplementary Table 1.

Dyspnea, cough, and discomfort were the most frequently reported symptoms of pleural and pericardial effusions, whereas abdominal distension, abdominal pain, and edema were the most widely recognized symptoms of ascites.

### Response

A total of 400 and 81 injections were administered for 85 patients. The average number of injections into the pericardial cavity was two, while that into pleural and intraperitoneal cavities were three to four. Only one patient with T cell lymphoblastic lymphoma received the maximum seven pleural injections.

Among the 85 patients, half had bloody drainage fluid, and tumor exfoliated cells were detected in the drainage fluid from 31 patients. The injections generally had limited toxicity, and only 11 patients developed a moderate fever on the first day of injection. No patient developed respiratory distress related to IL-2 and DEX injection therapy. Simultaneously, no allergic reaction or catheter-related infection occurred.

### Outcome

In this study, patients with solid tumors were mainly treated according to the protocols from COG or International Society of Pediatric Oncology group (SIOP )[10–16], whereas those with lymphoma were mainly treated in line with BFM protocols [17–20]. The vast majority of patients in the effusion group achieved met the criteria for early stopping (no more than 2 times injection) of the IL-2 and DEX injection. Even though one patient received seven injections altogether. No recurrence of pleural effusion, ascites, or pericardial effusion was noticed.

**Fig. 1:**
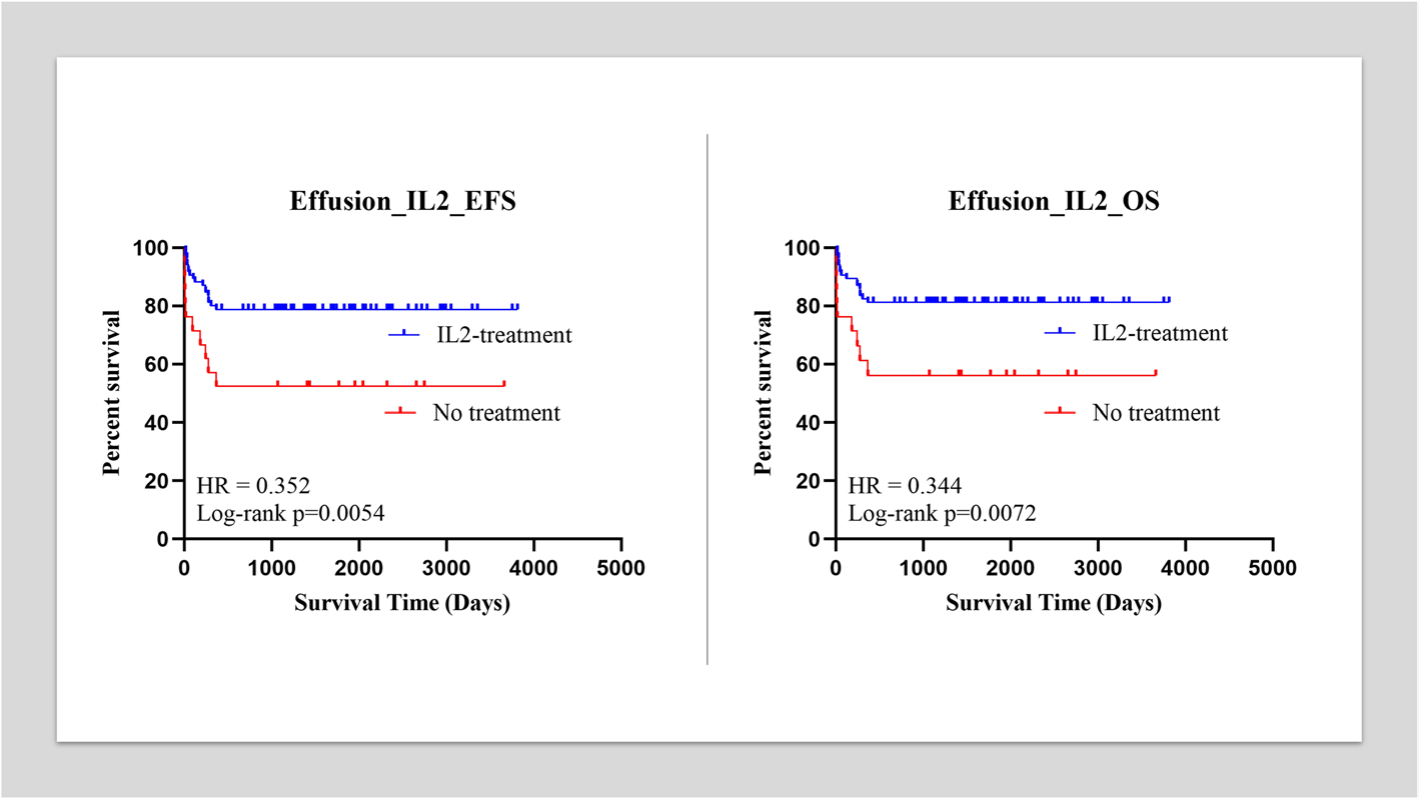
A: The Kaplan Meier analysis demonstrated significant differences between the effusion group and control group for lymphoma patients in EFS (*p* < 0.01). The hazard ratio was 0.191 for EFS. A: The Kaplan Meier analysis demonstrated significant differences between the effusion group and control group for lymphoma patients in OS (*p* < 0.01). The hazard ratio was 0.161 for OS

Among patients in the effusion group, four with lymphoma died of disease progression, and two had relapsed disease (including three with T cell lymphoblastic lymphoma, one with DLBCL, one with BL, and one with ALCL). One of the two patients with relapsed disease died, while the other with ALCL achieved SD after crizotinib treatment [21]. Four with solid tumor died due to disease progression, and eight got relapsed diseases (including three with RMS, one with NB, three with PPB, one with Ewing’s sarcoma, one with HB, and one with PNET). Seven of the eight patients with relapsed disease died, while the other with HB achieved secondary CR after irinotecan treatment [22]. Among patients in the control group, two with lymphoma died of disease progression, and two had relapsed disease (including one with T cell lymphoblastic lymphoma, two with DLBCL, and one with ALCL). Both patients with relapsed disease died. Two with solid tumors died due to disease progression, and four got relapsed diseases (including three with RMS, one with NB, one with Ewing’s sarcoma, and one with PNET). Three patients with relapsed disease died, while the other with NB achieved PR after irinotecan treatment and alive with tumor. The five-year OS was 81.18% (95%CI, 72.69 to 89.66%) in the effusion group and 57.14% (95%CI, 34.1 to 80.2%) in the control group. The five-year EFS was 78% (95%CI, 69.96 to 87.69%) in the effusion group and 52.38% (95%CI, 29.09 to 76.68%) in the control group. The Kaplan Meier analysis showed a significant difference between the two groups, with *p* < 0.01 for EFS and OS. HR = 0.344 (95%CI, 0.12 to 0.99) between OS and 0.352 (95%CI, 0.13 to 0.96) between EFS (Fig. 1). The mean effusion control time (met the criteria for stopping) for the effusion group was 5.76 days (95%CI, 5.34 to 6.19 days), while for the control group was 18.3 days (95%CI, 15.94 to 20.72 days), which had statistical difference (*p* < 0.01).

**Fig. 2:**
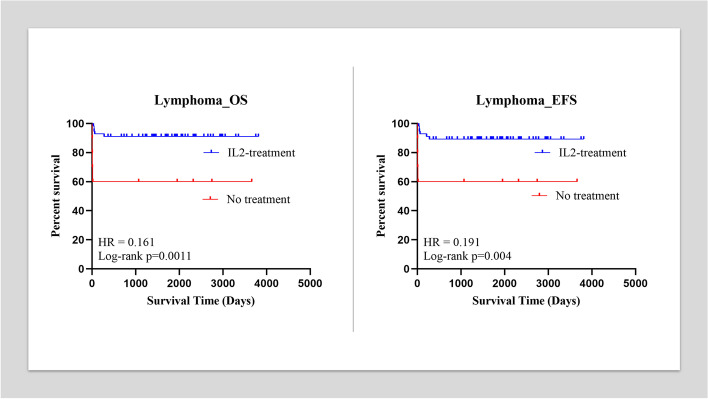
A: The Kaplan Meier analysis demonstrated no differences between the effusion group and control group for solid tumor patients in EFS (*p* > 0.05). A: The Kaplan Meier analysis demonstrated no differences between the effusion group and control group for solid tumor patients in OS (*p* > 0.05)

**Fig. 3:**
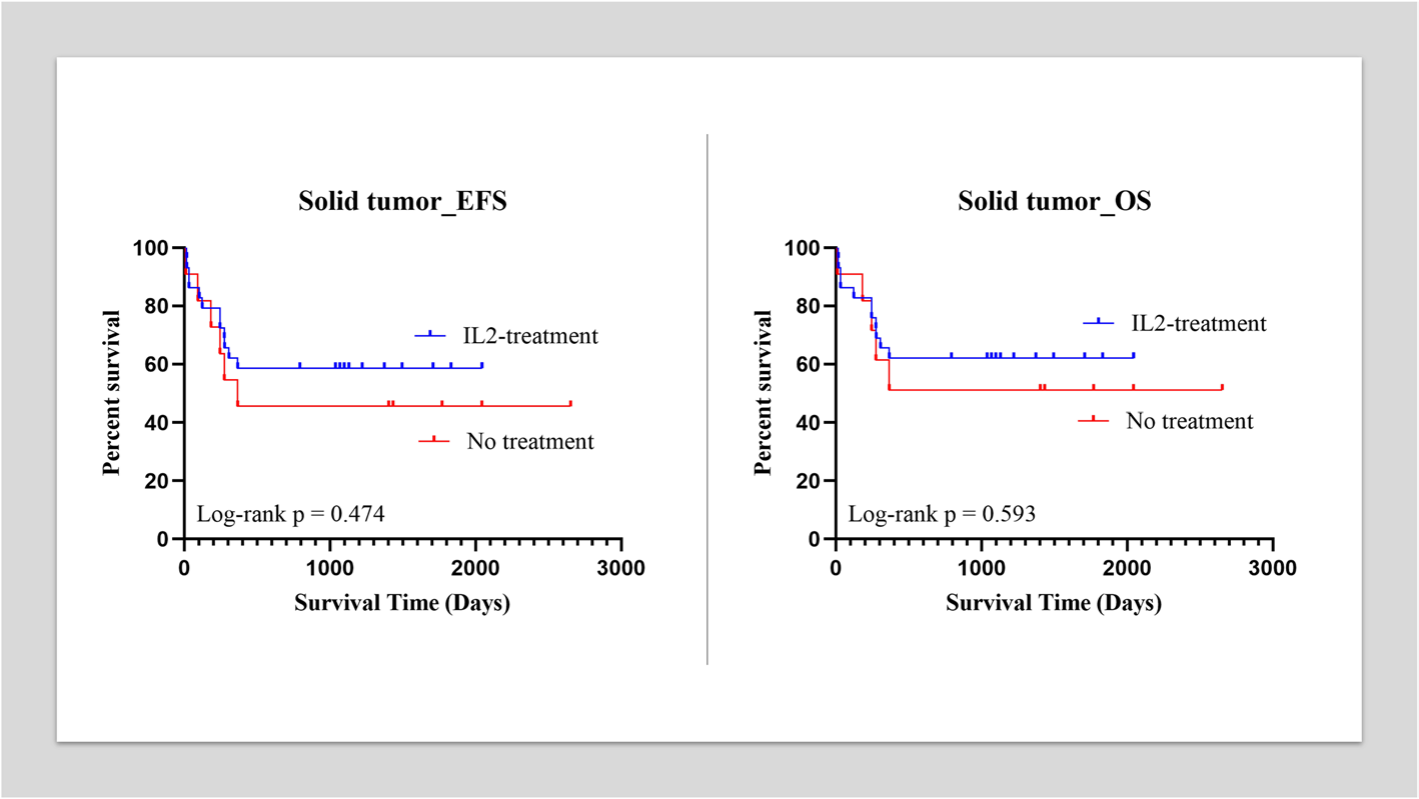
A: The Kaplan Meier analysis demonstrated significant differences between the effusion group and control group for pediatric cancer patients in EFS (*p* < 0.01). The hazard ratio was 0.352 for EFS. A: The Kaplan Meier analysis demonstrated significant differences between the effusion group and control group for pediatric cancer patients in OS (*p* < 0.01). The hazard ratio was 0.344 for OS

When we separated lymphoma with solid tumor, the five-year EFS and five-year OS for patients with lymphoma in effusion group were 89.3% (95%CI, 80.9 to 97.6%) and 91.1% (95%CI, 83.4 to 98.8%). While the five-year EFS and five-year OS for patients with lymphoma in control group were 60% (95%CI, 23.1 to 96.9%). The Kaplan Meier analysis demonstrated significant differences between the two groups with both of which *p* < 0.01. When we calculated the hazard ratio (HR), we found that it was 0.191 (95%CI, 0.03 to 1.35) for EFS and 0.161 (95%CI, 0.02 to 1.26) for OS (Fig. 2). For patients in the effusion group with solid tumors, the five-year EFS and five-year OS were 62.1% (95%CI, 43.3 to 80.9%) and 65.5% (95%CI, 47.1 to 83.9%), respectively. For patients in the control group with solid tumor, the five-year EFS and five-year OS were 45.5% (95%CI, 10.4 to 80.5%) and 54.5% (95%CI, 19.5 to 89.6%), respectively. The Kaplan Meier analysis showed no statistical difference between the two groups with both of which *p* > 0.05 (Fig. 3).

## Discussion

Several reports exist on the small size of pediatric patients with pleural effusion or ascites [2, 23, 24]. However, no existing study has been conducted to investigate the role of IL-2 and DEX in pediatric cancer patients with malignant pleural effusion, ascetics, and pericardial effusion. It has been well recognized that IL-2 plays a vital role in activating and maintaining specific and nonspecific immune responses [25]. IL-2 can induce activated natural killer cells and enhance antibody-dependent cellular cytotoxicity [26]. As such, IL-2 injection is applied in treating adult tumors [27, 28].

Remarkably, we found that DEX administration combined with IL-2 via thoracic, intraperitoneal, or pericardial injection quickly resolved the fluid and immediately relieved the discomfort of patients. Moreover, no recurrence of pleural effusion, ascites, or pericardial effusion was observed in our patients. Since this study is a retrospective study, the exact contribution of IL-2 can not be ascertained given the co-administration of DEX, which is a known therapeutic agent in lymphomas. However, the combination plays an important role in treating malignant effusions. Initially, we administered IL-2 and DEX without obtaining a pathological diagnosis to save the patient’s life when imaging examination revealed a potentially malignant tumor associated with pleural effusion, ascites, or pericardial effusion, especially in critically ill children. Our treatment is effective, as all symptoms, including chest pain and dyspnea, improved to varying degrees in affected children. The mean effusion control time for the effusion group was short while compared to the control group, which significantly differed. Although tumor-exfoliated cells were only detected in 31 patients, there was no misdiagnosis, and this procedure has become our regular treatment model.

Pleural effusion, ascites, and pericardial effusion are the possible signs of tumor spread, indicating the contamination of pleural space or abdominal cavity; thus, they are often considered negative prognostic factors [3, 24]. However, as reported in the study on NB patients from St. Jude Children’s Research Hospital, no difference is found in the survival related to a pleural effusion [2].

Our retrospective study included many tumors, so it was difficult to determine the tumor prognosis from survival. As such, the whole cohort of patients split by whether IL-2 and DEX were given or not. As we expected, the five-year EFS and five-year OS for patients in the effusion group was much higher than that in the control group. There was a statistical difference, and HR was < 1, indicating that IL-2 therapy is a protective factor for survival.

The NHL-BFM90 study reports a 90% EFS rate for patients with T-cell lymphoblastic lymphoma and a 93.9% three-year EFS for those with mature B cell lymphoma. In our study, the five-year EFS in the effusion group with lymphoma was 89.3%, comparable to those reported in other studies but not in the control group. As such, our study showed that with the appropriate treatment, pleural effusion, ascites, and pericardial effusion were not poor prognostic factors.

Nowadays, the 5-year OS for patients with pediatric solid tumors ranges from 50 to 80% [12, 29, 30]. Our five-year OS was slightly lower than the average level, which might be because that the number of patients in the solid tumor group was relatively small. Moreover, type III PPB is highly aggressive neoplasm with very poor 5 yrs progression free survival, ranged from 33% [31] to 42% [16]. Since three children were diagnosed with type III PPB in the effusion group while there was no PPB patient in the control group, and all three patients died. That might adversely affect the prognosis of the effusion group.

It is known that IL-2 administration is associated with numerous side effects, and there is evidence that increased doses of IL-2 lead to increased toxicity [32]. Several dosage regimens, including high intravenous doses (720,000 or 600,000 international units/kg), have been applied for obtaining the maximum therapeutic benefit [27]. At our hospital, the recommended dosage of IL-2 is 1 million IU/ m2/ time (maximum dose 10.0 × 106 IU). Our study suggested that IL-2 injection following this dose was well tolerated and highly safe. The possible mechanism of IL-2 in treating adult pleural effusion is that IL-2 increases the numbers of CD3 + T cells and NK cells in the pleural space and enhances the immune response, thus reducing the incidence of pleural effusion. However, the mechanism of IL-2 in treating pediatric cancers remains unknown. Most pediatric cancers arise from embryonal cells that are distinctly different from epithelial cells, and the immune response itself is also markedly different between adults and children. Consequently, the low mutational burden and relative lack of neoantigen expression are among the defining traits of pediatric cancers, which have limited their immune targeting susceptibility [33].

To sum up, this retrospective research demonstrates that thoracic, intraperitoneal injection or pericardial injection of DEX plus IL-2 is an effective and highly safe treatment for pediatric cancers with pleural effusion, ascites, and pericardial effusion. Nevertheless, there are several limitations of our study. This is a retrospective study, and the sample size (specially for the control group) is quite small. Besides, the co-administration of IL-2 and DEX makes it difficult to conclude which one of them was effective. Further randomized trials are warranted to provide more real evidence to evaluate the efficacy of IL-2 in treating pediatric patients.

## Supplementary Information

**Additional file 1.**

## Data Availability

Patient’s data were available in medical records room of the first hospital of Jilin university. The datasets generated and/or analysed during the current study are not publicly available due to they are files in medical records room in our hospital, but are available from the corresponding author on reasonable request.

## Abbreviations

IL-2: Interleukin-2
DEX: Dexamethasone
EFS: Event free survival
OS: Overall survival
NB: Neuroblastoma
HB: Hepatoblastoma
COG: Children’s Oncology Group
SE: Small effusion
ME: Moderate effusion
CT: Computer tomography
LE: Large effusion
G1: Grade 1
G2: Grade 2
G3: Grade 3
S: Small
M: Moderate
L: Large
MRI: Magnetic resonance imaging
CR: Complete response
PR: Partial response
PD: Progressive disease
SD: Stable disease
PNET: Primitive neuroectodermal tumor
PPB: Pediatric pneumoblastoma
DLBCL: Diffuse large B-cell lymphoma
BL: Burkitt’s lymphoma
ALCL: Anaplastic large cell lymphoma
RMS: Rhabdomyosarcoma
NaCl: Sodium chloride injection
SIOP: Society of Pediatric Oncology group
